# Supplementary material for: The interactome of intact mitochondria by cross-linking mass spectrometry provides evidence for coexisting respiratory supercomplexes
Source: Mol Cell Proteomics. 2017 Dec 8;17(2):216–32. doi: 10.1074/mcp.RA117.000470 (PMC5795388; doi:10.1074/mcp.RA117.000470)
Supplement: Supplemental Data [file supp_RA117.000470_133922_0_supp_23978_fzffwf.zip › spectra_annotation/mito_spectra_annotation/54-1-11-1-3-1.pdf]

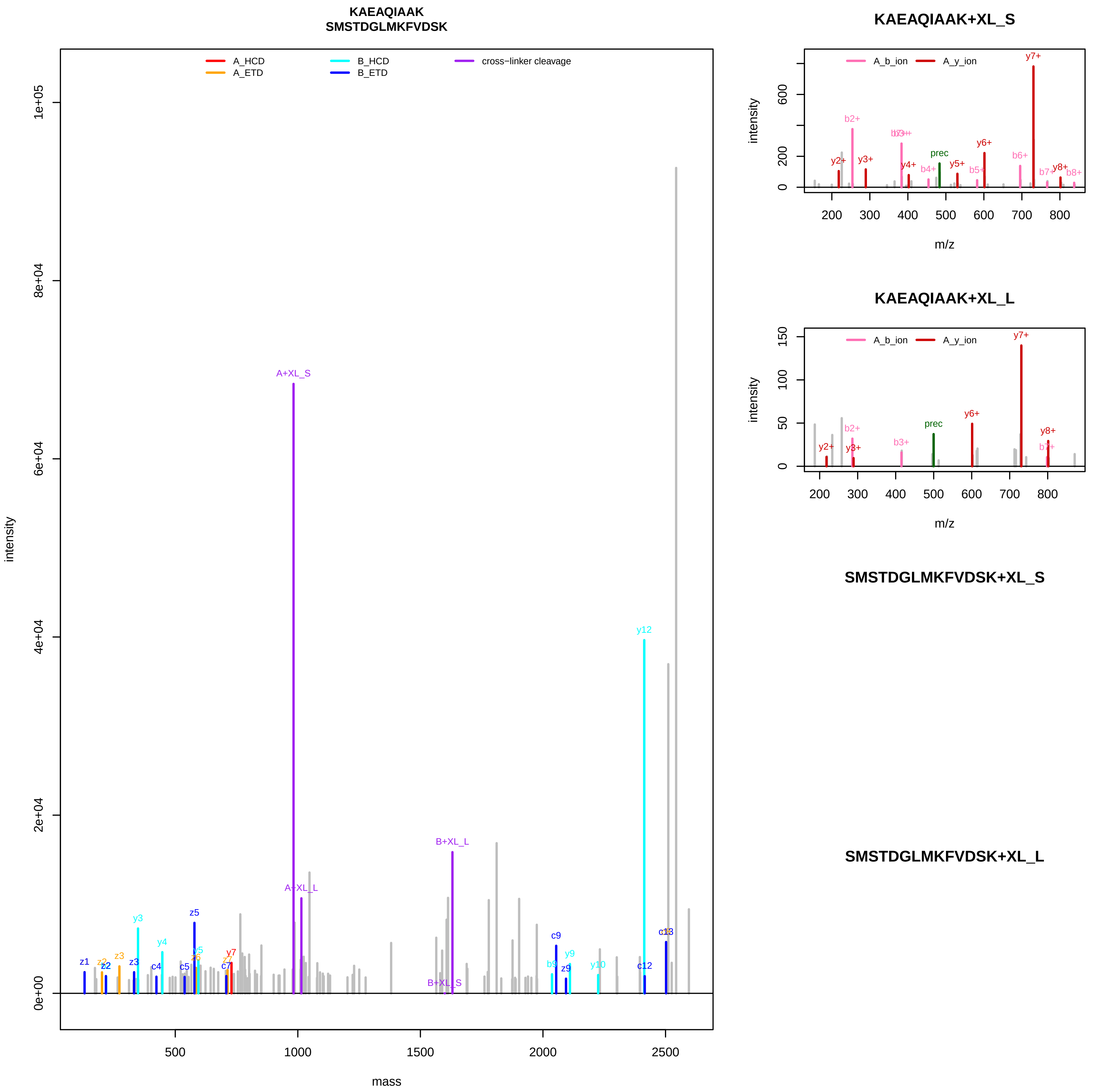

KAEAQIAAK
SMSTDGLMKFVDSK
A_HCD
A_ETD
B_HCD
B_ETD
cross−linker cleavage
intensity
mass
A+XL_S
A+XL_L
B+XL_L
B+XL_S
y12
c13
c12
y10
c9
y9
b9
z9
y3
y4
y5
z5
c4
c5
c7
y7
z1
z2
z3
KAEAQIAAK+XL_S
A_b_ion
A_y_ion
prec
m/z
KAEAQIAAK+XL_L
SMSTDGLMKFVDSK+XL_S
SMSTDGLMKFVDSK+XL_L
